# Supplementary material for: Using national virtual maternal death reviews to improve the quality of care during Pregnancy, labour and birth, and Postpartum in Tanzania
Source: PLoS One. 2026 Mar 18;21(3):e0344858. doi: 10.1371/journal.pone.0344858 (PMC12998800; doi:10.1371/journal.pone.0344858)
Supplement: S2 File — (DOCX) [file pone.0344858.s002.docx]

**Modifiable factors as captured from VMDR Reviews(2022-2023)**

| S/N | Themes(Factors) | Examples of specific gap identified during the review |
| --- | --- | --- |
| 1 | Equipment and supplies | Stock-out of magnesium sulphate |
|  |  | Lack of oxygen cylinders |
|  |  | Lack of cardiopulmonary monitors |
|  |  | Lack of suction machines |
|  |  | Lack/Non-functional vacuum extraction sets |
|  |  | Lack of antihypertensives |
|  |  | Lack of calcium gluconate |
|  |  | Lack of uterotonics eg Misoprostol |
|  |  | Stock-outs of IV Tranexamic acid |
|  |  | Lack of ultrasound machines |
|  |  | Stock-out of IV crystalloids |
|  |  | Stock-out of antibiotics |
|  |  | Lack of dialysis machine |
|  |  | Lack of ventilators |
|  |  | Lack/Non-functional Haematological/Serum biochemistry machine |
|  |  | Lack/stock-out of bacteria culture and sensitivity media and plates respectively |
|  |  | Lack/Stock out of Unfractionated Heparin |
|  |  | Lack of Non-pneumatic Anti-shock Garment (NASG) |
|  |  | Stock-out of laboratory reagents |
| 2 | Anesthesia issues | Accidental intra-thecal injection of Tranxamic acid |
|  |  | Stock of spinal needles |
|  |  | Lack of anaesthesia machine |
|  |  | Stock of general anaesthetic agents |
|  |  | Inappropriate choice of anaesthesia method |
|  |  | Inappropriate choice of anaesthesia agent |
|  |  | Stock out of Regional Anaesthetical agents |
|  |  | Lack of endotrachel intubation skills |
|  |  | Lack/Non-functional Laryngoscope sets |
|  |  | Inadequate duration of training of anaethetits |
|  |  | Lack of routine supervision of anaesthesia service at facility level |
|  |  | Limited number of trained anaethetits |
|  |  | Lack of reliable oxygen supply systems |
|  |  | Presence of only one anaethesia practitioner during surgeries |
|  |  | Lack of pre-anaethetic visit |
|  |  | Lack/non-functional surgical beds |
|  |  | Inadequate fluid pre-loading prior to Spinal anaesthesia |
|  |  | Lack of anaethesia monitoring during surgeries |
|  |  | Delay in recognitation of anaesthesia complications |
|  |  | Lack of skills in management of anaesthesia complications |
| 3 | Lack of blood | Inadequate blood replacement |
|  |  | Lack of whole blood |
|  |  | Lack of platelets |
|  |  | Lack of fresh frozen plasma |
|  |  | Lack of of SOPs on blood transfusion |
|  |  | Lack of blood storage refregarators |
|  |  | Failure to manage blood transfusion reactions |
|  |  | Stock-out of blood bags and blood giving sets |
|  |  | Stock-out of giving sets |
|  |  | Lack of knowledge on Massive Transfusion protocl |
|  |  | Lack of packed red-blood cells |
| 4 | Antenatal care factors | Lack of BP machine at Antenatal Clinic |
|  |  | Lack of cuvettes for checking Haemoglobin level |
|  |  | Lack of adequate counselling at ANC |
|  |  | Failure to recognize danger signs |
|  |  | Delayed referral to high level facility for further assessment and management |
|  |  | Non-adherence to counselling |
|  |  | Late ANC Booking |
| 5 | Surgical issues | Lack of skills in making approrpite uterine incision for caesarean section |
|  |  | Inadequate skills in chosing appropriate suture material for caesarean section repairs |
|  |  | Lack of skills in fetal extraction for cesarean sections perfomed in deep tranvserse arrest |
|  |  | Lack of skills in perform sub-total hysterectomy |
|  |  | Lack of skills to peform Uterine Compression sutures |
|  |  | Attempts to perform myomectomy during emergency CS procedure |
|  |  | Lack of use of WHO safe surgery checklist |
| 6 | Leadership and accountabiliy | Lack of staff/ organization at the facility |
|  |  | Failure to monitor supplies and medicines stocks |
|  |  | Delaye in identifying/responding to facility functionality gaps |
|  |  | Ignoring emergency calls when on duty |
|  |  | Lack of commitment to clinical care routines eg ward rounds |
|  |  | Lack of equipment preventive Maintanance plans |
|  |  | Failure to budget for ambulance fuel |
|  |  | Lack of routine supervision to subordinates |
| 7 | Practice &attitudes | Lack of team-work in management of complications |
|  |  | Lack/delay of consultation to senior practitioners when managing complications |
|  |  | Delayed response after consultation to manage complications |
|  |  | Clinician on emergency call roaster to ignore spending the night at the hospital |
|  |  | Lack/delayed inter-departmental consultations in tertiary hospital |
|  |  | Failure of documenting client information in case files |
|  |  | Non-adherence to Guidelines and Standard Operating Procedures |
|  |  | Failure to conduct adhere to standard post-operative monitoring |
|  |  | Filling clinical case history and examination findings without actually assessing the patient |
| 8 | Clinical Skills | Lack of labour diagnosis skills |
|  |  | lack of identification of abnormal labour findings in a partography |
|  |  | Lack of skills in using partograph for labour monitoring |
|  |  | Lack of basic pelvic assessment skills |
|  |  | Inappropriate actions for abnormal findings on obstetric assessment |
|  |  | Inability to detect and manage hypovolemic shock |
|  |  | Lack of skills to manage Septic Shock |
|  |  | Delayed diagnosis of PPH |
|  |  | Failure to achieve hemostasis during emergency caesarean section |
|  |  | Unjustified decisions to perform caesarean section |
|  |  | Lack of skills in breech delivery |
|  |  | Lack of skills in management of shoulder dystocia |
|  |  | Lack of skills in perfoming episiotomy |
|  |  | Lack of skills in perfoming Vacuum assisted vaginal delivery |
|  |  | Lack of skills in Management of PPH |
|  |  | Lack of skills in management of puerperal sepsis |
|  |  | Lack of skills in Management of abruptio placenta |
|  |  | Lack of skills on management of severe anaemia |
|  |  | Lack of skills on Management of Heart Failure |
|  |  | Lack of skills in management of Pre-eclampsia/Eclampsia |
