# Supplementary material for: Using national virtual maternal death reviews to improve the quality of care during Pregnancy, labour and birth, and Postpartum in Tanzania
Source: PLoS One. 2026 Mar 18;21(3):e0344858. doi: 10.1371/journal.pone.0344858 (PMC12998800; doi:10.1371/journal.pone.0344858)
Supplement: Attachment — Submitted filename: PONE-D-25-09586.docx [file pone.0344858.s004.docx]

PLOS ONE

Using national virtual maternal death reviews to improve the quality of care during

Pregnancy, labour and birth, and postpartum in Tanzania

--Manuscript Draft--

| Manuscript Number: | PONE-D-25-09586 |
| --- | --- |
| Article Type: | Research Article |
| Full Title: | Using national virtual maternal death reviews to improve the quality of care during Pregnancy, labour and birth, and postpartum in Tanzania |
| Short Title: | Using national virtual maternal death reviews to improve the quality of care. A lesson from Tanzania |
| Corresponding Author: | Ahmad Mohamed Makuwani, MD, MMed OBGY  Ministry of Health  Dodoma, TANZANIA, UNITED REPUBLIC OF |
| Keywords: | maternal death surveillance and response, maternal deaths reviews, virtual reviews, maternal mortality, quality of care, innovation in death reviews. |
| Abstract: | Background: Maternal and Perinatal Death Surveillance and Response (MPDSR) is an intervention recommended by WHO to improve care and, hence, reduce maternal and perinatal deaths. In collaboration with professional associations, Tanzania commenced daily online maternal death reviews (VMDRs) in December 2021 to improve the quality of reviews and accountability. We present the process and results of implementing the VMDR system in 2022-2023.  Methods: A retrospective descriptive study of VMDRs was conducted in 2022-2023 using a mixed-methods approach. This paper presents quantitative and qualitative data analyses done in Excel using the national monthly maternal death notification system and the VMDR.  Results: 369 VMDRs were conducted, and 687 maternal deaths were reviewed. Reviews observed that 49% of deaths were caused by obstetric hemorrhage, 16% by hypertensive disorders, 10% by anesthesia complications, 6% by sepsis, and 3% by abortion. Contributing factors included lack of competency (82%), suboptimal practice and attitude (69%), issues of leadership and accountability (42%), inadequate surgical care (37%), inadequate antenatal care (34%), lack of blood (32%), poor anesthesia practices (28%), and lack of equipment and supplies (24%).  The VMDR improved response, exemplified by one of the district hospitals making anesthetic supplies and devices available, specialty doctors starting to take the night shifts and improving key leaders at the national, regional and district level participation in the reviews.  VMDR enabled leaders' participation, understanding of challenges, and value for money in generating evidence. However, strong leadership is needed to maintain confidentiality in line with existing culture and norms.  Conclusion: The VMDRs improved the quality of reviews and accountability of healthcare service delivery. Tanzania has sustained VMDR through high acceptability and participation from leaders and experts. The role of VMDRs and related outputs must be periodically assessed for fidelity and feasibility in social reactions emanating from the political environment and health system decision-making process. |
| Order of Authors: | Ahmad Mohamed Makuwani, MD, MMed OBGY |
|  | Sunday Alfred Dominico, MD, MMed |
|  | Secilia Kapalata Ngweshemi, PhD |
|  | Golden Mwakibo Masika, PhD |
|  | Rose Mpembeni, PhD |
|  | Charles Ameh, PHD |
|  | Mzee Masumbuko Nassoro, MD, MMed |
|  | Phineas Sospeter, MD, MMed |
|  |  |

|  | Habib Ismail, MPH |
| --- | --- |
|  | Tumaini Nagu, PhD |
|  | Matilda Ngarina |
|  | Edwin Lugazia, MD, MMed |
|  | Pius Muzzazzi, MD, MMed |
|  | Beatrice Simwalike, PhD |
|  | Florina Serbanescu, PhD |
| Additional Information: |  |
| Question | Response |
| Financial Disclosure  Enter a financial disclosure statement that describes the sources of funding for the work included in this submission. Review the [submission guidelines](http://journals.plos.org/plosone/s/submission-guidelines#loc-financial-disclosure-statement) for detailed requirements. View published research articles from [PLOS ONE](https://journals.plos.org/plosone/) for specific examples.  This statement is required for submission and will appear in the published article if the submission is accepted. Please make sure it is accurate. | The author(s) received no specific funding for this work. |
| Funded studies Enter a statement with the following details: - Initials of the authors who received each award - Grant numbers awarded to each author - The full name of each funder - URL of each funder website - Did the sponsors or funders play any role in the study design, data collection and analysis, decision to publish, or preparation of the manuscript? |  |
| Did you receive funding for this work? |  |
| Competing Interests  Use the instructions below to enter a competing interest statement for this submission. On behalf of all authors, disclose any [competing interests](http://journals.plos.org/plosone/s/competing-interests) that could be perceived to bias this work—acknowledging all financial support and any other relevant financial or non- | One author from US CDC supported data analysis and set up of the manuscript |

| financial competing interests.  This statement is required for submission and will appear in the published article if the submission is accepted. Please make sure it is accurate and that any funding sources listed in your Funding Information later in the submission form are also declared in your Financial Disclosure statement.  View published research articles from [PLOS ONE](http://journals.plos.org/plosone/) for specific examples. |  |
| --- | --- |
| NO authors have competing interests Enter: The authors have declared that no competing interests exist. Authors with competing interests Enter competing interest details beginning with this statement: I have read the journal's policy and the authors of this manuscript have the following competing interests: [insert competing interests here] |  |
| * typeset |  |
| Ethics Statement  Enter an ethics statement for this submission. This statement is required if the study involved:   - Human participants - Human specimens or tissue - Vertebrate animals or cephalopods - Vertebrate embryos or tissues - Field research   Write "N/A" if the submission does not require an ethics statement.  General guidance is provided below.  Consult the [submission guidelines](http://journals.plos.org/plosone/s/submission-guidelines#loc-guidelines-for-specific-study-types) for | Data collection on maternal deaths and the review process are part of routine quality improvement efforts. The data on maternal death reviews are stored and achieved at the Ministry of Health—Department of Reproductive, Maternal, and Child Health Services. These data do not bear the deceased's name and are kept confidential.  These data were achieved and hence did not require individual clearance.  The Permission to publish these results was granted by the University of Dodoma  (UDOM) Institutional Research Review Committee (IRREC) and approved by the National Health Research Ethics Sub-Committee (NatHREC) of the National Institute for Medical Research (NIMR) with reference number MA.84/261/67/16 after seeking clearance from the Ministry of Health. |

detailed instructions.

Make sure that all

information entered here is included in the

Methods section of the manuscript.

Format for specific study types

Human Subject Research (involving human

participants and/or tissue)

Give the name of the institutional review

board or ethics committee that approved the

study

•

Include the approval number and/or a

statement indicating approval of this

research

•

Indicate the form of consent obtained

(

written/oral) or the reason that consent was

not obtained (e.g. the data were analyzed

anonymously)

•

Animal Research (involving vertebrate

animals, embryos or tissues)

Provide the name of the Institutional Animal

Care and Use Committee (IACUC) or other

relevant ethics board that reviewed the

study protocol, and indicate whether they

approved this research or granted a formal

waiver of ethical approval

•

Include an approval number if one was

obtained

•

If the study involved

non-human primates

,

add

additional details

about animal welfare

and steps taken to ameliorate suffering

•

If anesthesia, euthanasia, or any kind of

animal sacrifice is part of the study, include

briefly which substances and/or methods

were applied

•

Field Research

Include the following details if this study

involves the collection of plant, animal, or

other materials from a natural setting:

Field permit number

•

Name of the institution or relevant body that

granted permission

•

Data Availability

Authors are required to make all data

underlying the findings described fully

available, without restriction, and from the

time of publication. PLOS allows rare

Yes - all data are fully available without restriction

| exceptions to address legal and ethical concerns. See the [PLOS Data Policy](http://journals.plos.org/plosone/s/data-availability) and [FAQ](http://journals.plos.org/plosone/s/data-availability#loc-faqs-for-data-policy) for detailed information.  A Data Availability Statement describing where the data can be found is required at submission. Your answers to this question constitute the Data Availability Statement and will be published in the article, if accepted. |  |
| --- | --- |
| Important: Stating ‘data available on request from the author’ is not sufficient. If your data are only available upon request, select ‘No’ for the first question and explain your exceptional situation in the text box. |  |
| Do the authors confirm that all data underlying the findings described in their manuscript are fully available without restriction? |  |
| Describe where the data may be found in full sentences. If you are copying our sample text, replace any instances of XXX with the appropriate details.   - If the data are held or will be held in a public repository, include URLs, accession numbers or DOIs. If this information will only be available after acceptance, indicate this by ticking the box below. For example: All XXX files   are available from the XXX database (accession number(s) XXX, XXX.).   - If the data are all contained within the manuscript and/or Supporting Information files, enter the following: All relevant data are within the manuscript and its Supporting Information files. - If neither of these applies but you are able to provide details of access elsewhere, with or without limitations, please do so. For example:   Data cannot be shared publicly because of [XXX]. Data are available from the XXX Institutional Data Access / Ethics Committee (contact via XXX) for researchers who meet the criteria for | All data are available at Tanzania Ministry of Health, Department of Reproductive, Maternal and Child Health |

| access to confidential data.  The data underlying the results presented in the study are available from (include the name of the third party and contact information or URL).  • This text is appropriate if the data are owned by a third party and authors do not have permission to share the data.  * typeset |  |
| --- | --- |
| Additional data availability information: | Tick here if the URLs/accession numbers/DOIs will be available only after acceptance of the manuscript for publication so that we can ensure their inclusion before publication. |

Manuscript	[Click here to](https://www2.cloud.editorialmanager.com/pone/download.aspx?id=39592393&guid=acd6119e-db40-4890-a274-ad0421ffcdab&scheme=1)

[access/download;Manuscript;Tanzania_Manuscript_Virtue](https://www2.cloud.editorialmanager.com/pone/download.aspx?id=39592393&guid=acd6119e-db40-4890-a274-ad0421ffcdab&scheme=1)

# Using national virtual maternal death reviews to improve the quality of care during Pregnancy, labour and birth, and postpartum in Tanzania Authors: ^1^Ahmad Mohamed Makuwani, ^1^ Sunday Alfred Dominico, ^2^ Secilia

Kapalata Ngweshemi, ^2^ Golden Masika, ^3^ Rose Mpembeni, ^4^ Charles Ameh, ^1^

Mzee Masumbuko Nassoro, ^1^ Phineas Sospeter, ^1^ Habib Ismail, ^1^ Tumaini

Nagu, ^5^ Matilda Ngarina, ^6^ Edwin Lugazia, ^7^ Pius Muzzazzi, ^8^ Beatrice

Simwalike, ^9^ Florina Serbanescu

# Affiliations

1 Ministry of Health
2 University of Dodoma,
3 Muhimbili University of Health and Allied Sciences
4 Liverpool School of Tropical Medicine
5 Association of Obstetricians and Gynaecologists of Tanzania (AGOTA)
6 Society of Anaesthesiologists of Tanzania (SATA)
7 Paediatric Association of Tanzania (PAT)
8 Tanzania Midwives Association (TAMA)
9 U.S. Centers for Disease Control and Prevention, Division of Reproductive Health, Atlanta, GA, USA

On behalf of the Tanzania Virtual MPDSR Steering committee

**Corresponding Author:**

Ahmad Mohamed Makuwani, MD, MMED

Tanzania Ministry of Health, United Republic of Tanzania

Email: amakuwany@gmail.com

**Key Words:** *maternal death surveillance and response, maternal deaths reviews, virtual reviews, maternal mortality, quality of care, innovation in death reviews.*

# Introduction

Globally, an estimated 278,000 maternal deaths occurred in 2020. Despite accounting for only 10% of the world’s population(1), Sub-Saharan Africa contributes 70% of these deaths (202,000 maternal deaths) (2). Most maternal deaths in SSA occur during or within 24 hours of childbirth and postpartum period (3,4). An increasing number of births in the region occur in health facilities(5). Thus, improving the quality of delivery and post-delivery care in health facilities is a critical intervention to reduce maternal mortality and attain the Sustainable Developing Goal 3.1 of reducing the global maternal mortality ratio to no more than 70 maternal deaths per 100 000 live births by 2030 (6,7)

Maternal death reviews (MDR) are key interventions that aim to identify and address gaps within the health care system that contributed to the death of a woman during pregnancy, delivery or the first 42 days after delivery (8). Tanzania has been and continues to be among the top 10 countries with a high number of maternal deaths per year (2). In 2006, Tanzania implemented maternal and perinatal death reviews for the first time, as recommended by the World Health Organization (WHO) guidance *Beyond the Numbers, Reviewing Maternal Deaths, and Complications to Make Pregnancy Safer*(8)*.* The MDR process in Tanzania was scaled up into a surveillance system following the publication of the WHO Maternal Death Surveillance and Response (MDSR) technical guidance (9). This was captured in the first national guidelines issued in 2015 and revised in 2019 (10). As with other countries in the region, Tanzania has extended the surveillance system to include perinatal deaths (maternal and perinatal death surveillance and response or MPDSR). The MPDSR guidelines are intended to help eliminate preventable deaths in both facilities and communities by continuously documenting and analyzing the magnitude, trends, and impact of maternal mortality. The guidelines prioritize a focus on strengthening accountability in responding to gaps in health systems, investigation of community deaths through verbal autopsies, and use of the WHO applications of ICD-10 to deaths during pregnancy, childbirth, and the puerperium (ICD-MM) and deaths during the perinatal period (ICD-PM) (11,12,13). The guidelines provide ground rules for conducting death reviews, which include maintaining an atmosphere of “no shame, no blame, no name,” respecting the confidentiality of the discussion and accepting criticisms from peers to improve clinical care.

Tanzania MPDSR guidelines are fully institutionalized at the community, health facility, district, regional, and national levels. For a meaningful critical MDR process, highly skilled clinicians organized in health facility committees must be present during the review. These professionals are generally available in hospitals (mainly regional, zonal and national), often in towns and cities. Several assessments of the implementation of the national MPDSR guidelines indicated delays and suboptimal quality in conducting reviews in lower-level health facilities (14,15). Delays and suboptimal quality reviews were ~~parts~~partly the result of a lack of commitment among health managers, time constraints, and limited training of reviewers (14–18). Sub-optimal reviews led to incorrect assignment of causes of death, weak root-cause analysis (RCA) to identify gaps in the quality of care, and a failure to develop appropriate action plans that include specific, measurable, attainable, realistic, and timebound (SMART) recommendations (19).

Experts recommend that MDRs be institutionalized within the Ministry of Health (MoH) to provide oversight, policy guidance, and support to ensure a functional and effective process (9,17,20). As a response to the COVID-19 pandemic, the MoH instituted limited physical travel to review meetings to mitigate the spread of the virus. This led to the adoption of virtual social networking applications such as Zoom, WhatsApp, Microsoft Teams, and Google Meet to facilitate technical interactions and support clinical decision-making for junior healthcare providers.

Aiming to improve the quality of reviews and accountability of healthcare service delivery, the MoH, in collaboration with professional associations, commenced daily online independent expert virtual maternal death ~~review~~reviews (VMDR) in December 2021. This paper describes the process, results, successes, and lessons learnt from a two-year experience (January 2022 to December 2023) of virtual MDRs (VMDRs) coordinated by MoH.

**Methods**

**Design**

This is a quantitative and qualitative study.

# Settings and Study Population

The VMDR sessions were implemented nationally by reviewing maternal deaths reported from 1^st^ January 2022 to 31^st^ December 2023 in the 26 Tanzania Mainland regions, district councils and health facilities. Commencing in April 2024, these data became accessible for analysis. All reviewed deaths met the WHO standard definition— death of a woman while pregnant or within 42 days of termination of pregnancy, irrespective of the duration and the site of the pregnancy, from any cause related to or aggravated by the pregnancy or its management, but not from accidental or incidental causes (11). Deaths were included if they were classified as maternal deaths and had adequate documentation from reviews already conducted by the respective facilities.

# Conceptualisation and process of VMDR

While virtual reviews were conceptualised by the “no shame, no blame, no name” and confidentiality principles, their development has been informed by the “comfort zone” concept proposed by Judith Baldwick in 1991 (21). The comfort zone was defined as “a behavioural state within which a person operates in an anxiety-neutral condition, using a limited set of behaviours to deliver a steady level of performance, usually without a sense of risk.” The author proposes that “anxiety improves performance until a certain optimum level of arousal has been reached. Beyond that point, performance deteriorates as higher levels of anxiety are attained.” Adopters of the “comfort zone” concept in managing performance suggest that individuals or institutions need to move out of the their comfort zone and reach an “optimal anxiety” to maximise performance; too much anxiety, however, will decrease performance. In moving toward conceptualizing VMDRs, the MoH was aware that virtual sessions would increase the level of anxiety among health practitioners but ultimately decided that having expert interdisciplinary and multi-participatory peer review sessions increases the case ascertainment and identification of bottlenecks in the health system, fosters the development of SMART recommendations, and ultimately improves performance and quality of care in maternal health care settings (21).

The design and approach of the VMDR were agreed upon through consultative meetings of technical staff at the MoH, the President’s Office of Regional Administration and Local Government Authorities (PO-RALG), and experts from relevant professional associations. A secretariat of MPDSR experts at the Divisions of Reproductive, Maternal, and Child Health Services and Nursing and Midwifery Services of MoH coordinated reviews. The MoH created an online WhatsApp group forum for regional Reproductive and Child Health Coordinators (RCHCo) to notify maternal deaths that occurred in their regions. It also created a WhatsApp expert group for the national VMDR steering, which included volunteering maternal health experts from the MoH, Association of Gynaecologists and Obstetricians Tanzania, Tanzania Midwives Association, and Society of Anaesthesiologists in Tanzania. This group, in rotation, provides a chair and co-chair for the VMDR sessions.

# Virtual maternal death case review process

The VMDRs are daily Zoom sessions organised by the MoH in collaboration with the President’s Office of Regional Administration and Local Government Authorities (PO-RALG) and volunteering experts from relevant professional associations. The participation of experts is coordinated through the WhatsApp VMDR steering committee. Maternal death reviews are scheduled daily from Monday through Thursday (perinatal death reviews are scheduled on Fridays and are not described here). At the end of each month, MoH prepares the VMDR schedule that assigns the region, chairs and coordinators for each review date for the coming month. Next, the MoH distributes the schedule to the 26 Tanzanian Mainland regions and a larger audience through the WhatsApp expert group. The schedule shows which region needs to prepare and share a case each day of the month. Lastly, the region responsible for presenting the case for a specific day selects a case for VMDR and submits the presentation and any supportive documents 2-3 days before their designated VMDR session. Each case selected by the region has been previously reviewed at the health facility and/or district level. The sessions are recorded and documented. The documentation of each case is completed by the regional reproductive and child health coordinator from the presenting region. This documentation is completed before and during the meeting using a VMDR tool developed by MoH and validated by steering committee members.

The MoH coordinates the sessions and is chaired by national and subnational experts in obstetrics, midwifery, and anaesthesiology cadres with experience in MPDSR. Attendance comprises healthcare professionals, program managers at the facility, district, regional, and national levels, researchers, and invited guests. Attendance may range from 100 to over 300 participants. Each VMDR meeting lasts about 2 hours on average. Zoom licensing and internet costs are covered with funding from MoH and support from Jhpiego and Americares.

The link to each Zoom meeting is shared exclusively within formal professional groups consisting of healthcare providers and managers from medical, nursing, midwifery, pharmaceutical, and allied sciences. At the beginning of the review, the chair calls a meeting to order and instructs participants to register. Each participant must register with a full name, professional body (or board) registration number, and affiliation institution or health facility. Five minutes after the meeting, the chair instructs removing any participant with no identifiable registration.

Each meeting starts with the chair reminding the code of conduct that reaffirms the principles of no name, no blame, no shame, and confidentiality (10). Next, the presenting region updates the audience on actions taken by the region following the preceding VMDR hosted by the region. This is followed by a presentation from a health facility representative from the presenting region of a case summary for the current-day case, the probable cause of death, medical and non-medical factors that may have contributed to the death, any identified gaps in care, and potential responses. Anonymized documents collected from the health facility and local review committee and the 2019 MoH guidance serve as the basis for preparing the case summary. Information about the deceased and the attending healthcare providers is deidentified during the case presentation using abbreviations. The chair facilitates questions and answers that are aimed at reaching a consensus on the cause of death and performs a root-cause analysis (RCA) of the medical and non-medical factors that contributed to the death (22). During the case discussion, all microphones must be muted except those of the chair and contributors invited to speak by the chair.

Through an ~~iterative~~ interactive process, the VMDR participants identify clinical bottlenecks that led to death, gaps in the health systems and ~~decision making~~ decision-making, and actions to be taken to prevent future occurrences of similar events. The “Three Delays” framework (23) may organise the identified gaps. However, given that 81% of women give birth in health facilities in Tanzania (24), the VMDRs focus more on discussing and addressing the gaps in health care and referrals (delay 3) and less on delays at the individual and community levels.

After discussing the case, the presenter summarises the recommendations agreed upon during the VMDR and an action plan to implement these recommendations. The action plan, final cause of death, and medical and non-medical contributing factors are completed in the case documentation and submitted to the MoH. This information is collected using a VMDR case documentation tool, a Microsoft Word document template developed by MoH, and validated by the steering committee members (Annex 1). The documentation is completed manually by the Regional Nursing Officer/ Reproductive and Child Health Coordinator from the respective presenting region and shared with the MoH and the regional health management team.

No information from the review may be used for reprimand or litigation.

# Annex 1: VMDR case documentation template

Attendance at the VMDR is mandatory for the regional medical officer, district medical officer, medical officer-in-charge, and other members from respective regional and council health management teams responsible for supervising the presenting health facility. The review ensures the participation of MoH experts, professional councils and associations, and all healthcare providers. Some VMDRs receive a booster of attendance when the Minister for Health, Deputy Minister of Health, Permanent Secretary, or Chief Medical Officer announce they plan to attend.

# Data analysis

The data from each VMDR case documentation and the MPDSR monthly notification forms were entered into an Excel spreadsheet by the MoH personnel. Quantitative data were summarised using simple descriptive variables, such as age, parity, cause of death, and place of death, and presented as tables and charts. Case summaries and virtual meeting recordings were transcribed and coded using “Atlas.ti” software to conduct a deductive content analysis of the identified final causes of deaths. These modifiable factors contributed to the deaths, and recommendations were made to address them. Causes of death were classified using the WHO ICDMM grouping (12). The multitude of modifiable factors identified through VMDRs was classified into 8 categories, including clinical skills, practice and attitudes, leadership and accountability, ANC factors, provision of safe anaesthesia, surgical care issues, availability and use of blood and blood products, and availability of medicines and supplies (Annex 2). The gaps were tallied in an Excel sheet to establish how often they occurred across the reviewed deaths. Recommendations were synthesized by the level of health system responsible for their implementation.

# Ethical clearance

Data collection on maternal deaths and the review process are part of routine quality improvement efforts. The data on maternal death reviews are stored and ~~achieved~~archived at the Ministry of Health—Department of Reproductive, Maternal, and Child Health Services. These data do not bear ~~the~~ deceased's name and are kept confidential. These data were ~~achieved~~archived and hence did not require individual clearance.

The Permission to publish these results was granted by the University of Dodoma (UDOM) Institutional Research Review Committee (IRREC) and approved by the National Health Research Ethics Sub-Committee (NatHREC) of the National Institute for Medical Research (NIMR) with reference number MA.84/261/67/16 after seeking clearance from the Ministry of Health.

# Results

A total of 369 VMDRs were conducted, and 687 maternal deaths that occurred from January 2022 to December 2023 were virtually reviewed. Reviewed cases were selected from maternal deaths that occurred in zonal referral hospitals (n= 151; 22%), regional referral hospitals (n=159; 23%), district hospitals (n=179; 26%), health centres (n=136; 20%), dispensaries (n=42; 6%), and communities (n=20; 3%) (data not shown). Almost one-half of the 3,041 deaths notified to the MoH were from 7 regions (Dar es Salaam, Mwanza, Morogoro, Kigoma, Mtwara, Mbeya, and Tabora) (Table 1). Almost one-fourth (22.6%) of notified maternal deaths in 2022-2023 have been reviewed through VMDRs. The percentage of VMDR-reviewed deaths ranged from 11.3% in Dar es Salaam to 59.7% in Pwani region. Due to the rotating schedule of regions that are asked to present to the VMDR sessions, regions with fewer maternal deaths tend to have a higher percentage of their deaths reviewed virtually.

# Table 1: Number of notified maternal deaths and proportion of virtually reviewed maternal deaths by region in 2022–2023, Tanzania Mainland

| S/N | Region | No. of MDs notified to MOH | No. of cases reviewed virtually | % MD of  reviewed virtually |
| --- | --- | --- | --- | --- |
| 1. | Dar es Salaam | 382 | 43 | 11.3% |
| 2. | Mwanza | 309 | 52 | 16.8% |
| 3. | Morogoro | 204 | 31 | 15.2% |
| 4. | Kigoma | 178 | 31 | 17.4% |
| 5. | Mtwara | 151 | 36 | 23.8% |
| 6. | Mbeya | 140 | 39 | 27.9% |
| 7. | Tabora | 132 | 22 | 16.7% |
| 8. | Dodoma | 123 | 20 | 16.3% |
| 9. | Geita | 112 | 22 | 19.6% |
| 10. | Singida | 111 | 25 | 22.5% |
| 11. | Arusha | 101 | 22 | 21.8% |
| 12. | Kagera | 98 | 24 | 24.5% |
| 13. | Mara | 95 | 16 | 16.8% |
| 14. | Shinyanga | 94 | 23 | 24.5% |
| 15. | Rukwa | 88 | 26 | 29.5% |
| 16. | Manyara | 83 | 25 | 30.1% |
| 17. | Kilimanjaro | 76 | 24 | 31.6% |
| 18. | Pwani | 72 | 43 | 59.7% |
| 19. | Songwe | 71 | 26 | 36.6% |
| 20. | Lindi | 70 | 21 | 30.0% |
| 21. | Katavi | 69 | 19 | 27.5% |
| 22. | Iringa | 67 | 29 | 43.3% |
| 23. | Simiyu | 62 | 22 | 35.5% |
| 24. | Tanga | 59 | 20 | 33.9% |
| 25. | Ruvuma | 49 | 15 | 30.6% |
| 26. | Njombe | 45 | 11 | 24.4% |
|  | Overall | 3,041 | 687 | 22.6% |

The mean age of the 687 women who died of maternal causes and were VMDR-reviewed was 29.5 years, with a range of 15 to 50 years; 9% of women were less than 20 years old at the time of death. Nearly one-third

(32%) were grand multiparas (para 5 or higher), followed by primigravida (21%) (data not shown).

Of the 687 maternal deaths VMDR-reviewed, 90% were due to direct obstetric causes, and 9% were due to indirect causes. The leading direct causes were obstetric haemorrhage (49%), hypertensive disorders of pregnancy (16%), anaesthesia-related complications (10%), puerperal sepsis (6%), obstetric embolism (4%), and abortion-related complications (3%). The leading indirect obstetric causes were anaemia (5%), malaria (2.6%), and preexisting cardiac conditions (1.5%). The cause of death was not established in 1% of cases due to inadequate information during the review. (Figure 1).

# Figure 1: Distribution of causes of deaths in 687 virtually reviewed maternal deaths in 2022–2023, Tanzania Mainland

The leading non-medical factors contributing to the maternal deaths reviewed through VMDRs were related to insufficient clinical skills (82%), healthcare providers' attitudes (69%), suboptimal leadership and accountability (42%), inadequate surgical care (37%), inadequate antenatal care (34%), lack of blood (32%), poor anaesthesia practices (28%), and lack of equipment and supplies (24%). (Figure 2) These causes often overlapped, each case having multiple contributing factors.

# Figure 2. Main clinical and non-clinical modifiable factors in 687 virtually reviewed maternal deaths in 2022–2023, Tanzania Mainland

The VMDRs recommended specific actions (responses) to address avoidable factors related to each maternal death. Most agreed actions were related to 1) increased capacity of staff to manage obstetric complications that included improving surgical skills, improving consultations and mentorship (e.g., tertiary hospitals to improve multi-disciplinary consultations), and improving labour and postpartum monitoring; 2) adherence to the use of guidelines and standard operating procedures; and 3) increasing blood collection, processing, distribution and storage systems. This went hand in hand with the responsibilities of Regional Health Management Teams (RHMTs) and Council Health Management Teams (CHMTs) to ensure the functionality of the health system at primary healthcare levels, including the community.

In response to the VMDRs, RHMTs and CHMTs set up expert teams to build capacity based on identified gaps. Examples of the actions taken by the expert teams included safeguarding correct anaesthesia practices at one of the district hospitals, ensuring that specialist doctors in one of the zonal referral hospitals started a full-time night shift, connecting regional doctors to mentors in a zonal referral hospital to brush competencies; relocation of an anaesthesia machine to another health facility; and commencement of onsite mentorship to address key identified dysfunctions. The VMDRs managed to reinforce adherence to standards of care in the management of intrauterine foetal deaths, use of misoprostol for induction of labour, removal of retained products of conception, and use of low molecular weight heparin to prevent and treat obstetric thromboembolism.

At the national level, the MoH, professional associations, and partners have intensified resource mobilisation and partnerships, creating a system for healthcare capacity building. This is shown by developing a national MNCH mentorship guide and supporting clinical attachments of healthcare providers from lower-level facilities to high-level facilities. The Government has established mentorship in health facilities and commenced a virtual perinatal death review process and a weekly virtual Continuing Medical Education Forum (Table 2).

# Table 2: National Response on VMDR Data

| S/N | Issues | Responses |
| --- | --- | --- |
| National level | | |
| 1. | Mismatch in understanding between policymakers and implementers on overriding challenges in maternal health care | Policymakers have the same understanding as implementers that challenges on competence and leadership are key interventions for the health sector for now. |
|  |  | High-level national and Ministry leaders owned results from RCA that over 80% of current maternal deaths are attributed to issues of competence, practice, attitude, and leadership. |
| 2. | Lack of strong data-backed investment case to | More financial resources have been mobilized and allocated to address the competence gap, hire human |

|  | advocate for financial resources in maternal healthcare | resources for health (HRH), and procure lifesaving commodities and equipment. |
| --- | --- | --- |
| 3. | Limited quantities and quality of HRH in maternal healthcare | MOH started a mentorship plan where specialists from referral health go to primary health care facilities to work and mentor staff for at least two weeks. |
|  |  | Instituted a plan to have a panel of trainers for each region to support primary-level HFs. |
|  |  | More investment has been allocated to train service anaesthetists, anaesthesiologists, midwives, and obstetricians. |
| 4. | Inadequate readiness of service delivery system to manage common obstetric complications due to lack of providers’ competency | Commencement of weekly virtual CME sessions to improve knowledge and skills in managing common obstetric complications. A total of 42 sessions were conducted from 2022 - 2023 |
|  |  | Supporting experts’ teams to conduct visits to regions and facilities for follow-up on the implementation status of action plans agreed upon during virtual reviews |
|  |  | Supporting experts’ teams to visit regions and facilities with high burden/recurrences of specific causes of death for a detailed targeted clinical audit and RCA. |
|  |  | Development of the National Maternal, Newborn, and Child Health Mentorship guidelines and strategy to standardize capacity building for HCPs. |
|  |  | Design and commence implementation of structured clinical mentorship programs to build HCPs' capacity for managing common obstetric complications. |
|  |  | Sharing the findings and conducting high-level panel discussions with experts from medical training institutions on challenges of skills, practice, attitude, leadership, and stewardship of health service delivery |
| 5. | Persistence of Postpartum Haemorrhage as the cause of Maternal Death | Commencement of review of national EmONC Job aids, learning resource Package to adopt WHO 2023 Recommendations on Management of PPH, revised to accommodate findings of a groundbreaking multicountry and multicentric trial on early detection and management of PPH |
| 6. |  | Improved use of uterotonic guidelines in the induction and augmentation of labour, especially in |

|  | Full-strength use of technologies, medicines, and medical devices | preeclampsia/eclampsia, management of intrauterine foetal deaths, and postdate pregnancies. |
| --- | --- | --- |
|  |  | Increase in use of heparin in post caesarean section patients and other risk patients for venous thromboembolism features. |
| 7. | Persistent low quality of  MDRs | Conduct training of the first Batch of 30 National MPDSR trainers across 10 priority regions to enable them to cascade these skills to peers in their home regions and facilities |
| 8. | Lack of adherence to  Guidelines and Standard  Operating Procedures | Commenced revision of the National EmONC learning resource package and Job aids to accommodate recent Global updates e.g. E-motive Bundle in Management of PPH |
| Regional/Council level | | |
| 1. | Lack of healthcare providers' skills in the management of obstetric complications, including the performance of obstetric surgeries | Conduct clinical attachments of healthcare providers with limitations in skills to tertiary facilities for a duration of four to 12 weeks |
|  |  | Organize and support multi-disciplinary teams of experienced obstetricians/Gynaecologists to visit facilities for onsite mentorship on specific topics |
|  |  | Establishment and support of mobile emergency obstetric teams to promptly travel to respective facilities to attend emergencies |
| 2 | Limited number and quality of anaesthesia practitioners | Support tuition fees for clinical officers and nursemidwives to undertake one year of anaesthesia training at tertiary facilities |
|  |  | Redistribution of anaesthetists from District Hospitals to Health centres lacking/in a critical shortage of anaesthetists |
| 3 | Lack of functional ambulance | Repair of ambulance using district council financial resources |
| 4 | Limited quality of maternal death reviews | Commencement of virtual independent reviews at the regional level in some regions |
| 5 | Shortage of healthcare providers in newly  established CEmONC Health centres | Re-distribution/reallocation of healthcare providers from district hospitals with better quantities of HRH |
|  |  | Recruitment of new healthcare providers on short-term contracts using local financial resources |
| 6 | Inadequate quality of EmONC services at newly established CEmONC sites | Form and support a team of experienced practitioners from Tertiary facilities to conduct targeted clinical audits on care systems, caesarean sections, and nearmiss cases and respond accordingly to gaps identified |
| 7 | Inappropriate referral procedures, including a lack of skilled healthcare providers with equipment and supplies to support referrals | Ensure availability of appropriately skilled cadre with needed resuscitation equipment to escort clients during referrals to ensure continuity of care |
| Facility level | |  |
| 1. | Senior experts on Emergency roasters staying at home at night in public hospitals | Re-arrangements made, including creating an enabling environment for teams on call to stay at the facility throughout the night and promptly respond to emergencies |
| 2 | Failure to adhere to standard operating  procedures, guidelines, and SOPs | Printing, distribution, and orientation of healthcare providers on the use of the standard operating procedures and job aids |
| 3 | Lack of staff organization at the facilities, including work roasters and ward rounds | Re-organise staff through routine and emergency(oncall) duty roasters and commencement of routine daily ward rounds |
| 4 | Frequent stockouts of routine and emergency medicines and supplies | Designate providers responsible for forecasting, routine stock management, and re-stocking (early ordering) of commodities |

# Discussion

The VMDR of 687 cases revealed critical gaps in clinical care, with limited clinical skills playing a role in 82% of maternal deaths. At the same time, providers' suboptimal practices and attitudes contributed to 69% of the mismanagement of cases with obstetric complications. This is consistent with the findings from a systematic review of 81 studies conducted by Mannava, et al.—the majority (n=55, 68%) set in Africa - showing that maternal healthcare providers' beliefs, prejudices, and feelings of superiority could lead to negative attitudes that undermined patient health care and well-being (25). It is also consistent with a systematic review of avoidable factors in maternal and perinatal deaths in low-resource settings that identified substandard provision of care as the number one avoidable factor in 44 studies (26). Considering these findings, VMDRs prioritised responses that include continuing professional development for health workers and linkages to mentoring and support supervision, thus helping improve the quality of care and implementing a wide range of evidence-based interventions.

Lack of supplies, equipment and deficiencies in blood transfusion capacity have been identified as contributing factors to MDs in low- and middle-income countries (14,27–29). While only a quarter of VMDRs identified these factors, addressing these gaps has substantial costing and planning implications that require integration and coordination with health system strengthening actions at the regional and national levels.

The VMDRs in Tanzania benefited from wide acceptability, coordination and institutionalization. These were achieved through ensuring 1) multiprofessional participation with a focus on practical discussions of medical and non-medical factors contributing to maternal deaths and on formulating responses supported by close implementation plans at all levels; 2) a linkage with continuing medical education; and 3) integration within the national professional development framework. Smith, et al. (30) also reported that the MDR process is widely accepted and ongoing at the subnational level in many countries. Still, the process is not adequately institutionalized and lacks continuity from facility-based reviews to a continuous MDSR system that informs the wider health system. A qualitative study among health providers and health managers in Mtwara region, Tanzania, also showed broad support and acceptability of the MDR process but also important challenges, including insufficient provider training in conducting reviews, disconnect between reviews and quality improvement systems and organizational bottlenecks

(14).

The VMDRs improved leadership, participation of expert and healthcare providers and commitment to maternal health care - demonstrated by attendance of the Minister, Deputy Minister, Permanent Secretary and Chief Medical Officer, medical and nursing professional bodies, directors from MoH and PO-RALG, national, RHMTs and CHMTs, and leaders and members of professional associations. The VMDR improved the response of CHMTs and RHMTs to notification, review and implementation of action plans. Teams go to the health facilities and communities where maternal deaths have occurred and facilitate reviews. Findings from their reviews are quickly relayed to the districts, regions and the MoH for prompt responses. The participation of key stakeholders in the VMDRs, including professional associations, has shaped the clinical practice in health facilities.

The fundamental principles for conducting MDRs are well stipulated in the

WHO’s 2004 Beyond the Numbers publication and reiterated in its 2013 technical guidance for implementing MDSR (8,9). Conducting virtual reviews is not an approach mentioned by these guidelines; even the most recent WHO guide on MPDSR implementation (31) does not include VMDRs. The VMDR approach in Tanzania is the first example of continuous institutional utilization of VMDR. The adoption and continuous implementation to date have been possible due to the willingness of dedicated leaders, experts (specialists, general practitioners, nurse-midwives, paramedics) and policymakers at the district, regional and national levels to support a system that ensures a constructive dialogue and informed decision-making.

Only one published study, Okafor et al., documented the use of VMDR in a global maternal sepsis study involving deaths in 15 health facilities located in 11 LMICs (32)The study examined documentation on 13 virtually reviewed infection-related maternal deaths and 19 near-miss cases in 20 meetings conducted by international external reviewers. The review identified many modifiable factors organized using the 3-delay modal, which showed that most of those contributing factors to maternal deaths (n=71, 83%) were related to delays in receiving timely and appropriate care.

In implementing VMDR, Tanzania has upheld the principle of no name, no blame and no shame. However, this has been possible through strong chairmanship, rigorous vetting of participants, anonymized information and reminders of the importance of observing norms and core principles at the beginning of each review. While it has been possible to us, caution is recommended in other settings, as VMDR is thought to rely on social constructs to accommodate peers’ opinions without untoward reactions against the co-values of MPDSR (31).

The VMDR has changed an existing culture of shifting the focus from individual and community factors to the overall quality of healthcare services. This is due to three reasons. First, the VMDR sessions discuss modifiable factors related to timely and appropriate care at length. Secondly, almost all deaths that have been reviewed occurred in health facilities. Third, the approach uses RCA to establish system issues responsible for the death and recommends system-based interventions (33,34). The RCA is well aligned with the fact that deaths resulting from medical error exceed those associated with motor car accidents, breast cancer, or HIV/AIDS (35).

# Strengths and limitations

The distribution of VMDRs by cause is not representative. Still, it generally aligns with global, regional and national causes of MDs (36–38), where obstetric haemorrhage and pre-eclampsia are the leading causes. One exception is the high proportion of maternal deaths related to obstetric anaesthesia documented in VMDRs, which is several times higher than in the published literature. A recent systematic review found that anaesthesia accounted for an estimated 3.5% of direct maternal deaths in low- and ~~middleincome~~ middle income countries (39). Whereas the selection of cases submitted for virtual reviews may have influenced the per cent distribution of causes of maternal deaths, or this is the true magnitude of anaesthesia-related deaths in Tanzania remains unclear.

Conducting VMDR consistently and regularly may help key players in maternal health care and related disciplines overcome their comfort zones and provide quality care through improved competence, practice, and attitudes. The success of VMDRs relies on champions at the regional, subnational, and national levels, including professional associations, who are dedicated to the entire review process and to follow up on recommendations.

The quality of documents used in reviews may directly impact the recommendations from maternal death audits. Missing or incomplete patient information and data on the care received may lead to unsatisfactory conclusions on causes and circumstances of maternal deaths and failure to identify substandard care and corrective actions correctly (41–43). In VMDR, data abstraction is thorough, with clear event narration and documentation on the cause of death and underlying factors. If records are incomplete, an additional step is triggered via WhatsApp to reach out to all healthcare providers involved in the case management and complete missing information. This approach has enabled the generation of clear documentation on the cases that were reviewed and, in turn, enabled accurate descriptions of medical and non-medical causes that contributed to the death and improved the quality of recommendations. The same has influenced both the government and stakeholders to adopt the best approaches to improve the quality of care, focusing on improving healthcare providers' competency, practice and attitude through pre-service courses and in-service mentorship and supported supervision.

The selection of cases for VMDR is delegated to different regions. Therefore, the review team does not have control over the documentation and presentation of each case submitted for virtual review. Maternal deaths that are identified by regions have already been reviewed by health facilities and sometimes by district and regional committees. It is possible that the selection of the VMDR cases may be biased toward cases with complete medical information and documentation. Maternal deaths with incomplete or inadequate information or those whose care spanned over several wards or health facilities or required readmission may not be selected for VMDRs, which reduces the opportunity to learn from and formulate actions toward quality improvement targeting the most difficult cases. As VMDRs usually do not extend to maternal deaths outside health facilities, few community deaths were reviewed by the VMDR. Further, some facility MDR committees may not review maternal deaths due to indirect maternal causes, which may explain the low contribution of these causes among VMDR cases.

# CONCLUSION AND RECOMMENDATION

MPDSR(write in full) in low-resource settings is a complex process described in many kinds of literature, but there are still issues in implementation and maintenance. The WHO implementation guidelines allow for adaptability because the implementation and maintenance mechanisms will vary depending on the needs of a specific health system. The VMDR is an innovation that is possible and useful in settings with similar health systems and challenges, including the limited availability of experts to oversee and contribute to a comprehensive review process. Tanzania has managed to conduct and sustain VMDR through a diverse group acceptability and participation of leaders and experts through a sustained education of all stakeholders to uphold and maintain a key principle of review of ‘no blame, no shame’.

# ACKNOWLEDGEMENT

We thank our Ministers, Permanent Secretaries, Chief Medical Officer, Directors from MOH and PORALG, and professional bodies and associations for supporting VMDR. We sincerely thank the VMDR steering and coordination team for their tireless work to make this intervention run uninterrupted for three years from 2022 to date. The names these members are as follows; Felix Ambrose Bundala - Assistant Director (MPH), Saturin Manangwa - MOH (Nurse Midwife), Ziada Sellah - MOH Director Nurse

Midwife Services, Upendo Mamchony - MOH (Nurse Midwife), Cecilia Msafiri

- MOH (Nurse-Midwife specialist), Agness Mbio - MOH (Nurse Midwife),

Faraja Jacob Mgeni - MOH (Nurse Midwife), Jackline Ndanshau - MOH

(Nurse Midwife), Juma DaimonNyakina - Bukoba Regional Referral Hospital

(RRH) (OBGY), Paul John Sanka - Dodoma RRH (Nurse Midwife), Grace

Aziza Machenje - MOH (Nurse Midwife), Naibu Samweli Mkongwa - MOH (MPH), Ulimbakisye Yoram Macdonald - MOH (OBGY), Victoria Lyimo - MOH

(Nurse Midwife), Enock Mwantyala - MOH (MPH), Angela Leornard - MOH

(Paediatricaian), Rachel Greyshom Yangwa - MOH (Nurse Midwife), Ephraim

Kaphilimbi Shilla - MOH (Nurse Midwife), Fidea Obimbo - MOH (Nurse

Midwife), Naomi Chamhene - - MOH (Nurse Midwife), Tito Amosi Chaula -

Nkinga RH (OBSGY), Alfred Laison Mwakalebela - Iringa RRH (OBGY),

Edwin Rwebugisa Lugazia - MUHAS (Anaesthesiologist), Donald Micah

Maziku - Tosamaganga Hospital (OBSGY), George Rweyemamu Alcard -

Mbeya RRH (OBSGY), Elias Asiwelo Kaminyoge - Mbeya (Meta) Zonal

Hospital (OBGY), Chrisostom Clarence Lipingu - USAID-Afya Yangu/Jhpiego

(OBGY), Rita Elias Lyamuya - Morogoro RRH (OBGY) Ali Said - MUHAS

(OBGY), Elias Gregory Kweyamba - TTCIH (OBSGY), Berno Kasmir

Mwambe - Marie Stopes Hospital (OBGY), France John Rwegoshora - Mbeya

(Meta) Zonal Hospital - (OBGY), Titus Bonifas Mmasi - Monduli DC (OBGY),

Shedrack Andrew Magambo - UDOM Hospital (OBGY), Patrick Joseph

Kushoka - DCMC Hospital (OBSGY), John Deogratias Lawi - Iringa RRH

(OBGY), Matilda Michael Ngarina - MNH (OBS/GYN), Pendo Samwel Mlay - KCMC (OBGY), Emmanuel Imani Ngadaya - Tosamaganga Hospital (OBGY),

Chetan Pratapsinh Ramaiya - (OBGY), Nestory Andrew Mkenda - USAID-

Afya Yangu (OBGY), Naomi Amon Mwamanenge - Aghakan Hospital

(Neonatologist), Lucy Lawrence Mpayo - Neonatologist, Halima Mohamed

Kassim - Dodoma RRH (Paediatrician), Joyce Stephen Gimonge - Geita RRH

(Paediatrician), Emmanuel Rhubera Mafwiri - Njombe RRH (OBGY),

Georgina Balyorugulu - Kamanga Hospital (Paediatrician), Yustina Adam

Tizeba - Shinyanga RRH (Paediatrician), Tatu Seif Mbotoni - Iringa RRH

(Paediatrician), Castory Jerome Mwanga - Simiyu RRH (Paediatrician),

Beatrice Erastus Mwilike - MUHAS (Nurse midwife), Karoli Jerome Muna -

Kondoa DH (OBGY), Enock Hashim Dolli - Bukoba RRH (Paediatrician),

Mwanahawa Ramadhani Suba - Nkinga RH (Paediatrician), Amina Shomari

Shabani - Morogoro RRH (NurseMidwife), Happyphania Mathew - Kibaha TC

Hospital (Paediatrician), Jafari Bakari Lutavi - MNH (Nurse Midwife), Bahati

Katembo - Benjamin Mkapa Hospital (Nurse Midwife), Beatrice Bernard Kambuga - DIHAS Milembe (Nurse Midwife), Elizabeth Mkashabani - Paediatrician, Florentina Mashuda - CUHAS BMC (Paediatrician), Dismas

Matovelo - CUHAS BMC (OBGY), Monica Mumwi - Ndanda RH

(Paediatrician), Mary Sapali - Iringa RRH (Nurse Midwife), Nikolas Albert

Sagumo Chotta - Dodoma RRH (Paediatrician), Lucy Mabada - TAMA (Nurse

Midwife), Feddy Mwanga - TAMA (Nurse Midwife), Hawa Ngasongwa -

Morogoro RRH (Paediatrician) Elisamia Ngowi - Aghakan Hospital

(Paediatrician). Also, we would like to thank the Regional, Council, and Health Management Teams for their support in case identification, preparation, presentation, and follow-up of implementation of VMDR action plans. We thank everyone who participated and made input to VMDR from Tanzania and abroad. Without all of you, the VMDR would not be successful.

# References

1. United Nations Department of Economic and Social Affairs. World Population Prospects 2022 World Population Prospects 2022 Summary of Results [Internet]. 2022 [cited 2024 Jul 11]. Available from: population.un.org/wpp
2. WHO. https://data-unicef-org. 2023. UNICEF, United Nations Population Fund and The World Bank, Trends in Maternal Mortality: 2000 to 2020 WHO, Geneva.
3. Say L, Chou D, Gemmill A, Tunçalp Ö, Moller AB, Daniels J, et al. Global causes of maternal death: A WHO systematic analysis. Lancet Glob Health. 2014;2(6).
4. Kassebaum NJ, Bertozzi-Villa A, Coggeshall MS, Shackelford KA, Steiner C, Heuton KR, et al. Global, regional, and national levels and causes of maternal mortality during 1990-2013: A systematic analysis for the Global Burden of Disease Study 2013. The Lancet.

2014;384(9947).

5. United Nations Children Fund (UNICEF). Global Delivery Care Trends; 2015-2022. New York; 2022. Available from: https://data.unicef.org/topic/maternal-health/delivery-care/
6. United Nations. Transforming Our World: The 2030 Agenda for Sustainable Development. 2015. Available from: https://sdgs.un.org/publications/transforming-our-world-2030-agendasustainable-development-17981
7. Kruk ME, Gage AD, Arsenault C, Jordan K, Leslie HH, Roder-DeWan S, et al. High-quality health systems in the Sustainable Development Goals era: time for a revolution. Vol. 6, The Lancet Global Health. 2018.
8. World Health Organization. Reproductive Health and Research. Beyond the numbers: reviewing maternal deaths and complications to make pregnancy safer. World Health Organization; Geneva, Switzerland.

2004.

9. World Health Organization. Maternal death surveillance and response: technical guidance information for action to prevent maternal death. World Health Organization; Geneva, Switzerland. 2013. Available from: https://iris.who.int/bitstream/handle/10665/87340/9789241506083_eng. pdf?sequence=1
10. The United Republic of Tanzania Ministry of Health. Maternal and Perinatal Death Surveillance and Response Guidelines. MoH, Dodoma, Tanzania. 2019.
11. World Health Organization. ICD-10: International statistical classification of diseases and related health problems. 10th ed. WHO; Geneva Switzerland. 1992.
12. World Health Organization. The WHO application of ICD-10 to deaths during pregnancy, childbirth and the puerperium: IDC MM. World Health Organization; Geneva, Switzerland. 2012.
13. World Health Organization. The WHO application of ICD-10 to deaths during the perinatal period: ICD-PM. Geneva: World Health

Organization; 2016

14. Said A, Sirili N, Massawe S, Pembe AB, Hanson C, Malqvist M. Mismatched ambition, execution and outcomes: Implementing maternal death surveillance and response system in Mtwara region, Tanzania.

BMJ Glob Health. 2021 May 21;6(5).

15. Maternal and Child Survival Program. Assessment of Maternal and Perinatal Death Surveillance and Response (MPDSR) Implementation in Kagera and Mara Region, Tanzania. 2018. Available from: [https://mcsprogram.org/resource/assessment-of-maternal-andperinatal-death-surveillance-and-response-mpdsr-implementation-inkagera-and-mara-region-tanzania](https://mcsprogram.org/resource/assessment-of-maternal-and-perinatal-death-surveillance-and-response-mpdsr-implementation-in-kagera-and-mara-region-tanzania)
16. Kinney MV, Ajayi G, de Graft-Johnson J, Hill K, Khadka N, Om’Iniabohs

A, et al. “It might be a statistic to me, but every death matters.”: An assessment of facility-level maternal and perinatal death surveillance and response systems in four sub-Saharan African countries. PLoS One. 2020;15(12 December).

17. Kinney MV, Walugembe DR, Wanduru P, Waiswa P, George A. Maternal and perinatal death surveillance and response in low- And middle-income countries: A scoping review of implementation factors.

Vol. 36, Health Policy and Planning. Oxford University Press; 2021. p.

955–73.

18. Kashililika CJ, Moshi FV. Implementation of maternal and perinatal death surveillance and response system among health facilities in Morogoro Region: a descriptive cross-sectional study. BMC Health Serv Res. 2021;21(1).
19. Said A, Pembe AB, Massawe S, Hanson C, Malqvist M. Maternal death surveillance and response in Tanzania: comprehensiveness of narrative summaries and action points from maternal death reviews. BMC Health Serv Res. 2021 Dec 1;21(1).
20. Whiting-Collins L, Serbanescu F, Moller AB, Binzen S, Monet JP, Cresswell JA, et al. Maternal death surveillance and response system reports from 32 low-middle income countries, 2011–2020: What can we learn from the reports? Robinson J, editor. PLOS Global Public Health.

2024 Mar 5;4(3):e0002153. Available from: https://dx.plos.org/10.1371/journal.pgph.0002153

21. Bardwick JM. Danger in the comfort zone: from boardroom to mailroom-

-how to break the entitlement habit that’s killing American business.

New York, NY: Amacom, American Management Association. 1991.

22. Andersen, B., & Fagerhaug, T. Root cause analysis: Simplified tools and techniques. Milwaukee: ASQ Quality Press. 2000.
23. Thaddeus S, Maine D. Too far to walk: Maternal mortality in context.

Soc Sci Med. 1994 Apr 1;38(8):1091–110.

24. Ministry of Health (MoH) [Tanzania Mainland], Ministry of Health (MoH) [Zanzibar], National Bureau of Statistics (NBS), Office of the Chief Government Statistician (OCGS), and ICF. Tanzania Demographic and Health Survey and Malaria Indicator Survey 2022 Final Report. Dodoma, Tanzania and Dodoma, Tanzania, and Rockville, Maryland, USA. 2023.
25. Mannava P, Durrant K, Fisher J, Chersich M, Luchters S. Attitudes and behaviours of maternal health care providers in interactions with clients:

A systematic review. Global Health. 2015 Aug 15;11(1).

26. Merali HS, Lipsitz S, Hevelone N, Gawande AA et al. Audit-identified avoidable factors in maternal and perinatal deaths in low resource settings: A systematic review. BMC Pregnancy Childbirth. 2014;14(1).
27. Lewis G. Emerging lessons from the FIGO LOGIC initiative on maternal death and near-miss reviews. International Journal of Gynecology and Obstetrics. 2014;127(S1).
28. Endris AA, Tilahun T. Health system readiness to manage maternal death data and avail evidence for decision-making through the Maternal Death Surveillance System in Ethiopia, 2020. BMC Health Serv Res.

2023;23(1).

29. The United Republic of Tanzania Ministry of Health and Social Welfare.

Needs Assessment of Reproductive Maternal Newborn and Child Health in the Lake and Western Zones of Tanzania Technical Report.

2015.

30. Smith H, Ameh C, Roos N, Mathai M, van den Broek N. Implementing maternal death surveillance and response: A review of lessons from country case studies. BMC Pregnancy Childbirth. 2017 Jul 17;17(1).
31. World Health Organization. Maternal and Perinatal Death Surveillance and Response: Materials to Support Implementation. 2021.
32. Okafor O, Roos N, Abdosh AA, Adesina O, Alaoui Z, Romero WA, et al. International virtual confidential reviews of infection-related maternal deaths and near-miss in 11 low- and middle-income countries – case report series and suggested actions. BMC Pregnancy Childbirth. 2022 Dec 1;22(1).
33. Singh G, Patel RH, Boster J. Root Cause Analysis and Medical Error Prevention. StatPearls. 2021.
34. Kinney MV, Day LT, Palestra F, Biswas A, Jackson D, Roos N, et al. Overcoming blame culture: key strategies to catalyse maternal and perinatal death surveillance and response. Vol. 129, BJOG: An International Journal of Obstetrics and Gynaecology; 2022. p. 839–44.
35. Patient safety factsheet [Internet]. Available from:

https://www.who.int/news-room/fact-sheets/detail/patient-safety

36. Afnan-Holmes H, Magoma M, John T, et al. Tanzania's countdown to 2015: an analysis of two decades of progress and gaps for reproductive, maternal, newborn, and child health, to inform priorities for post-2015. Lancet Glob Health. 2015;3(7):e396-e409. doi:10.1016/S2214-109X(15)00059-5
37. Makuwani AM, Sospeter PF, Subi L, Nyamhagatta MA, et al. Baseline data on trend of maternal mortality in Tanzania using administrative data and its policy implication. 2018 report. Glob J Med Res. 2020:5–

12.

38. Musarandega R, Nyakura M, Machekano R, Pattinson R, Munjanja SP. Causes of maternal mortality in Sub-Saharan Africa: A systematic review of studies published from 2015 to 2020. J Glob Health. 2021;11.
39. Sobhy S, Zamora J, Dharmarajah K, et al. Anaesthesia-related maternal mortality in low-income and middle-income countries: a systematic

review and meta-analysis. *Lancet Glob Health*. 2016;4(5):e320-e327. doi:10.1016/S2214-109X(16)30003-1

40. Cahyanti RD, Widyawati W, Hakimi M. The reliability of maternal audit instruments to assign cause of death in maternal deaths review process: a systematic review and meta-analysis. BMC Pregnancy Childbirth. 2021 Dec 17;21(1):380.
41. Kao S, Chen L, Shi L, Weinrich MC. Underreporting and misclassification of maternal mortality in Taiwan. Acta Obstet Gynecol Scand. 1997 Aug 31;76(7):629–36.
42. Chandramohan D, Rodrigues LC, Maude GH, Hayes RJ. The validity of verbal autopsies for assessing the causes of institutional maternal death.

Stud Fam Plann. 1998 Dec;29(4):414–22.

Distribution of causes of deaths among 687 reviewed deaths	[Click here to access/download;Figure;Figure 1.docx](https://www2.cloud.editorialmanager.com/pone/download.aspx?id=39444823&guid=a935b1e1-76e8-49fe-ae77-d8f30a0d1f94&scheme=1)

*Figure 1. Distribution of causes of deaths in 687 virtually reviewed maternal deaths in 2022 - 2023, Tanzania Mainland*

Modifiable causes of deaths	[Click here to access/download;Figure;Figure 2.docx](https://www2.cloud.editorialmanager.com/pone/download.aspx?id=39444866&guid=4c1d6a15-87f7-419f-8467-7bd678c5635a&scheme=1)

*Figure 2. Main clinical and non-clinical modifiable factors in 687 virtually reviewed maternal deaths in 2022–2023, Tanzania*

*Mainland*
