# Supplementary material for: Using national virtual maternal death reviews to improve the quality of care during Pregnancy, labour and birth, and Postpartum in Tanzania
Source: PLoS One. 2026 Mar 18;21(3):e0344858. doi: 10.1371/journal.pone.0344858 (PMC12998800; doi:10.1371/journal.pone.0344858)
Supplement: Attachment — Submitted filename: Response to reviewers.doc [file pone.0344858.s007.doc]

May,29^th^ 2025

Dear Joyce Jebet Cheptum

Academic Editor

**PLOS ONE**

On behalf of all authors, we are grateful for the prompt feedback and comments from the editorial team and reviewers. Their insights have allowed us to enhance the paper’s substance and clarity. We have reviewed the statements on authors’ competing interests and have reconsidered that there is no competing interest. Furthermore, the data sharing statement has been revised as all the authors have consented to share all the data. Otherwise, we have made all the suggested changes to the paper and with this letter, we are submitting the revised Manuscript versions with and without track changes(clean). The point-by-point response on how we have addressed each of the issues is presented in the table below.

Table: Issues addressed in the Manuscript

| **S/N** | | **Reviewer/Editor** | **Comment** | **Response** |
| --- | --- | --- | --- | --- |
| 1. | | Editor | Manuscript meets PLOS ONE's style requirements, including those for file naming. | The font and headings in the Main Body and Title Page (including authorship) have been formatted according to PLOS ONE guidelines. |
| 2. | | Editor | Please confirm that competing interest declared on CDC author does not alter adherence to all PLOS ONE policies on sharing data and materials, by including the following statement: "This does not alter our adherence to PLOS ONE policies on sharing data and materials. | The competing interest note has been reviewed, and it is noted that no competing interest exists. |
|  | | Editor | Include your updated Competing Interests statement in your cover letter; we will change the online submission form on your behalf. | We have revised this section, and the competing interest note has been removed. There is no competing interest. |
| 3. | | Editor | When completing the data availability statement of the submission form, you indicated that you will make your data available on acceptance. We strongly recommend all authors decide on a data sharing plan before acceptance, as the process can be lengthy and hold up publication timelines. Please be assured that, once you have provided your new statement, the assessment of your exemption will not hold up the peer review process. | Authors have been consulted and have agreed to make all the data available for sharing when needed throughout the peer review and after acceptance.  We have also included supplementary data files 1 and 2, and captions added at the end of the Manuscript |
| 4. | | Editor | One of the noted authors is a group or consortium “Tanzania Virtual MPDSR Steering committee”.  In addition to naming the author group, please list the individual authors and affiliations within this group in the acknowledgments section of your manuscript. Please also indicate clearly a lead author for this group along with a contact email address. | Individual authors have been listed in the acknowledgement section  Name and  the email for the lead author for the group has been added |
| 5. | | Editor | We note that you have included the phrase “data not shown” in your manuscript. Unfortunately, this does not meet our data sharing requirements. PLOS does not permit references to inaccessible data. We require that authors provide all relevant data within the paper, Or, if the data are not a core part of the research being presented in your study, we ask that you remove the phrase that refers to these data. | The phrase with the data has been removed as it was found to have no additional information for the paper. |
| 6. | | Editor | Please amend your manuscript to include your abstract after the title page. | The amendment is done, and the abstract is included on the title page. |
| 7. | | Editor | Please review your reference list to ensure that it is complete and correct. If you have cited papers that have been retracted, please include the rationale for doing so in the manuscript text, or remove these references and replace them with relevant current references. Any changes to the reference list should be mentioned in the rebuttal letter that accompanies your revised manuscript | We have reviewed and changed the reference list, updating some references with the latest available literature. |
| 8. | | Editor | Beef up the methodology section, especially the study design. It is not adequate to just mention quantitative and qualitative | The methodology section, especially the study design, has been improved. |
| 9. | | Reviewer #1 | The manuscript is well-written with very few typographical errors (eg data "achieved" instead of "archived", "is" instead of "was"). These were highlighted in the text as "comments." | Thank you for the observation. The typo has been corrected |
| 10. | | Reviwer # 1 | The work addresses a very important intervention in obstetrics practice in line with WHO recommendations which is commendable. | Thank you very much |
| 11. | | Reviewer #2 | Thank you for asking me to review this research which is an excellent innovation and appropriate for MPDSR | Thank you very much |
| 12. | | Reviewer #2 | I wish to note the followings: 1. The title is apt | Thank you, this is noted. |
| 13. | | Reviewer #2 | 2. Discussion: The first two sentences are unnecessary repetition of results/findings already highlighted, hence should be rephrased. | The first paragraph has been rephrased in the Discussion section to highlight the purpose of the study and a summary of major findings |
| 14. | Reviewer #2 | | 3. Conclusion: MPDSR should be written in full. Acronyms even when previously defined are best avoided in starting a sentence. | Noted, and MPDSR written in full in the conclusion |
| 15. | Reviewer #2 | | 4. A few words were in British and some in American English. It is best to use one consistently in a manuscript. | Thank you, this has been noted and attended to |
| 16. | Reviewer #2 | | 5. I have added a few annotations to corrections These are minor corrections and the article can be accepted for publication afterwards. Thank you | Thank you, the corrections have been noted and accommodated. |
| 17. | Reviewer #3 | | Your manuscript holds significant value. Below are some suggestions to help enhance your research: | Thank you |
| 18. | Reviewer #3 | | 1. In the abstract, clearly articulate the necessity of the research. | The abstract has been revised to articulate the necessity of the study |
| 19. | Reviewer #3 | | 2. In the introduction, outline the innovative aspects of your research in the final paragraph. | The innovative aspects have been outlined in the last paragraph of the introduction |
| 20. | Reviewer #3 | | 3. At the conclusion of the discussion, incorporate suggestions for future studies. | The suggestion for future studies has been incorporated |
| 21. | General | | While revising your submission, please upload your figure files to the Preflight Analysis and Conversion Engine (PACE) digital diagnostic tool, [https://pacev2.apexcovantage.com/](https://track.editorialmanager.com/CL0/https:%2F%2Fpacev2.apexcovantage.com%2F/1/010f0195fa9cd204-2e13d4af-d0dd-40cc-b731-45fcffd58946-000000/Z40x1RAB6wDIyYc40FAVlDBhFJlJFMLzsBl9QTn_s4o=205). PACE helps ensure that figures meet PLOS requirements. To use PACE, you must first register as a user. Registration is free. Then, login and navigate to the UPLOAD tab, where you will find detailed instructions on how to use the tool. | Files uploaded and checked in PACE |

Thank you for reconsidering our manuscript for submission to your esteemed journal. We look forward to any further communication related to our changes.

Dr. Ahmad M. Makuwani,

**Corresponding author**
