# Supplementary material for: Using national virtual maternal death reviews to improve the quality of care during Pregnancy, labour and birth, and Postpartum in Tanzania
Source: PLoS One. 2026 Mar 18;21(3):e0344858. doi: 10.1371/journal.pone.0344858 (PMC12998800; doi:10.1371/journal.pone.0344858)
Supplement: Attachment — Submitted filename: Comments to Authors.docx [file pone.0344858.s006.docx]

Comments to Authors

1. Abstract: The study title does not tally well with the objective in the background and conclusion. From the abstract background and conclusion, your aim seems to be better maternal death review quality and accountability of healthcare delivery. Kindly see how you can either adjust the title or modify the aims and conclusion to speak to the title of the study.
2. On the Methods:
a) The tone of the entire paper seems to suggest that what you conducted was a retrospective review of VMDR records. Kindly clarify if the study was conducted prospectively or retrospectively. As this has implications. You indicate it was a mixed method study. What type of mixed methods was it (i.e convergent parallel design, explanatory sequential design, exploratory sequential design..). Since the reports based conclusion on what was discussed during the VMDR, what additional value did the transcribed data add? Did the conclusions and causes of death change? ..(In my view this was a retrospective review of VMDR records and not a mixed methods study). Kindly review mixed methods research and countercheck if your study meets the criteria.
b) You mentioned high acceptability: Acceptability is an implementation outcome I agree. But how did you measure acceptability in this particular study?
c) How did you measure participation from leaders and experts?
d) You also added fidelity and feasibility in the conclusion. These are also implementation outcomes. Kindly indicate in your methods section how all these outcomes indicated in the conclusion were measured.
3. Results: On Table 2, some of the responses are SAMRT eg “Commencement of weekly virtual CME sessions to improve knowledge and skills in managing common obstetric complications. A total of 42 sessions were conducted from 2022 – 2023”

However for some it is difficult to differentiate recommendations from what was actually done. See comments below

| S/N | Issues | Responses |
| --- | --- | --- |
| National level | | |
| 1. | Mismatch in understanding between policymakers and implementers on overriding challenges in maternal health care | Policymakers have the same understanding as implementers that challenges on competence and leadership are key interventions for the health sector for now. |
|  |  | High-level National and Ministry leaders owned the results from RCA that over 80% of current maternal deaths are attributed to issues of competence, practice, attitude, and leadership. |
| 2. | Lack of a strong data-backed investment case to advocate for financial resources in maternal healthcare | More financial resources have been mobilised and allocated to address the competence gap, hire human resources for health (HRH), and procure lifesaving commodities and equipment. |
| 3. | Limited quantities and quality of HRH in maternal healthcare | MOH started a mentorship plan where specialists from referral health go to primary health care facilities to work and mentor staff for at least two weeks. |
|  |  | Instituted a plan to have a panel of trainers for each region to support primary-level HFs. |
|  |  | More investment has been allocated to train service anaesthetists, anaesthesiologists, midwives, and obstetricians. |
| 4. | Inadequate readiness of the service delivery system to manage common obstetric complications due to a lack of providers’ competency | Commencement of weekly virtual CME sessions to improve knowledge and skills in managing common obstetric complications. A total of 42 sessions were conducted from 2022 - 2023 |
|  |  | Supporting experts’ teams to conduct visits to regions and facilities for follow-up on the implementation status of action plans agreed upon during virtual reviews. |
|  |  | Supporting experts’ teams to visit regions and facilities with high burden/recurrences of specific causes of death for a detailed targeted clinical audit and RCA. |
|  |  | Development of the National Maternal, Newborn, and Child Health Mentorship guidelines and strategy to standardise capacity building for HCPs. |
|  |  | Design and commence implementation of structured clinical mentorship programs to build HCPs' capacity for managing common obstetric complications. |
|  |  | Sharing the findings and conducting high-level panel discussions with experts from medical training institutions on challenges of skills, practice, attitude, leadership, and stewardship of health service delivery |
| 5. | Persistence of Postpartum Haemorrhage as the cause of Maternal Death | Commencement of review of national EmONC Job aids, learning resource Package to adopt WHO 2023 Recommendations on Management of PPH, revised to accommodate findings of a groundbreaking multi-country and multicentric trial on early detection and management of PPH |
| 6. | Full-strength use of technologies, medicines, and medical devices | Improved use of uterotonic guidelines in the induction and augmentation of labour, especially in preeclampsia/eclampsia, management of intrauterine foetal deaths, and postdate pregnancies. |
|  |  | Increase in use of heparin in post-caesarean section patients and other high-risk patients for venous thromboembolism features. |
| 7. | Persistent low quality of MDRs | Conduct training of the first Batch of 30 National MPDSR trainers across 10 priority regions to enable them to cascade these skills to peers in their home regions and facilities. |
| 8. | Lack of adherence to Guidelines and Standard Operating Procedures | Commenced revision of the National EmONC learning resource package and Job aids to accommodate recent Global updates, e.g. E-motive Bundle in Management of PPH |
| Regional/Council level | | |
| 1. | Lack of healthcare providers' skills in the management of obstetric complications, including the performance of obstetric surgeries | Conduct clinical attachments of healthcare providers with limitations in skills to tertiary facilities for a duration of four to 12 weeks. |
|  |  | Organise and support multi-disciplinary teams of experienced obstetricians/Gynaecologists to visit facilities for onsite mentorship on specific topics |
|  |  | Establishment and support of mobile emergency obstetric teams to promptly travel to respective facilities to attend emergencies |
| 2 | Limited number and quality of anaesthesia practitioners | Support tuition fees for clinical officers and nurse-midwives to undertake one year of anaesthesia training at tertiary facilities |
|  |  | Redistribution of anaesthetists from District Hospitals to Health centres lacking/in a critical shortage of anaesthetists. |
| 3 | Lack of a functional ambulance | Repair of the ambulance using the district council financial resources |
| 4 | Limited quality of maternal death reviews | Commencement of virtual independent reviews at the regional level in some regions |
| 5 | Shortage of healthcare providers in newly established CEmONC Health centres | Re-distribution/reallocation of healthcare providers from district hospitals with better quantities of HRH |
|  |  | Recruitment of new healthcare providers on short-term contracts using local financial resources |
| 6 | Inadequate quality of EmONC services at newly established CEmONC sites | Form and support a team of experienced practitioners from Tertiary facilities to conduct targeted clinical audits on care systems, caesarean sections, and near-miss cases and respond accordingly to gaps identified |
| 7 | Inappropriate referral procedures, including a lack of skilled healthcare providers with equipment and supplies to support referrals | Ensure availability of an appropriately skilled cadre with needed resuscitation equipment to escort clients during referrals to ensure continuity of care |
| Facility level | | |
| 1. | Senior experts on Emergency roasters are staying at home at night in public hospitals | Re-arrangements made, including creating an enabling environment for teams on call to stay at the facility throughout the night and promptly respond to emergencies |
| 2 | Failure to adhere to standard operating procedures, guidelines, and SOPs | Printing, distribution, and orientation of healthcare providers on the use of the standard operating procedures and job aids |
| 3 | Lack of staff organization at the facilities, including work rosters and ward rounds | Reorganise staff through routine and emergency(on-call) duty rosters and commencement of routine daily ward rounds. |
| 4 | Frequent stockouts of routine and emergency medicines and supplies | Designate providers responsible for forecasting, routine stock management, and re-stocking (early ordering) of commodities |

The comments apply for all the responses. Maybe you could separate recommendations from what has already been done

4. On leadership, you indicated “The VMDRs improved leadership, participation of expert and healthcare providers and commitment to maternal health care - demonstrated by attendance of the Minister, Deputy Minister, Permanent Secretary and Chief Medical Officer, medical and nursing professional bodies, directors from MoH and PO-RALG, national, RHMTs and CHMTs, and leaders and members of professional associations. The VMDR improved the response of CHMTS and RHMTS to notification, review and implementation of action plans”. In how many of the VDMR sessions were the Minister, Deputy minister and Permanent Secretary present. Do you have a baseline on their participation in MPDSR as a comparison….in short how did you come to the conclusion that VDMR improved leadership, participation of healthcare providers.
5. The Conclusion: Does not speak to the title. The issues of acceptability as indicated earlier in 2 above
