# Supplementary material for: Using national virtual maternal death reviews to improve the quality of care during Pregnancy, labour and birth, and Postpartum in Tanzania
Source: PLoS One. 2026 Mar 18;21(3):e0344858. doi: 10.1371/journal.pone.0344858 (PMC12998800; doi:10.1371/journal.pone.0344858)
Supplement: Attachment — Submitted filename: Response_to_reviewers_auresp_2.doc [file pone.0344858.s008.doc]

August 26^th^ 2025

Dear Joyce Jebet Cheptum

Academic Editor

**PLOS ONE**

Dear Editor,

On behalf of all co-authors, we thank you and the reviewers sincerely for your detailed review of our manuscript. Your constructive feedback has helped us refine the study and improve its clarity and methodological rigour. Below is our point-by-point response to the issues raised.

| S/N | Reviewers’ comment | Authors’ response |
| --- | --- | --- |
| 1 | **Abstract:** The study title does not tally well with the objective in the background and conclusion. From the abstract background and conclusion, your aim seems to be better maternal death review quality and accountability of healthcare delivery. Kindly see how you can either adjust the title or modify the aims and conclusion to speak to the title of the study. | The abstract and title have been revised to ensure consistency and alignment objective and methods of the manuscript |
| 2a | **On the Methods:**  The tone of the entire paper seems to suggest that what you conducted was a retrospective review of VMDR records. Kindly clarify if the study was conducted prospectively or retrospectively. As this has implications. You indicate it was a mixed method study. What type of mixed methods was it (i.e convergent parallel design, explanatory sequential design, exploratory sequential design..). Since the reports based conclusion on what was discussed during the VMDR, what additional value did the transcribed data add? Did the conclusions and causes of death change? ..(In my view this was a retrospective review of VMDR records and not a mixed methods study). Kindly review mixed methods research and countercheck if your study meets the criteria. | We agree with the reviewer’s observation. This study is a retrospective review of the VMDR sessions, with recommendations analyzed using a thematic narrative synthesis approach. The added value of the transcribed discussions was to provide context, nuance, and explanatory depth to the findings. While the quantitative data highlighted the leading medical causes of maternal and perinatal deaths, the transcripts revealed underlying health system and socio-cultural factors. These insights did not alter the final causes of death assigned by the VMDR committees, but they significantly enriched the interpretation and recommendations derived from the reviews. We updated the Methods section to clarify this distinction |
| 2b | You mentioned high acceptability: Acceptability is an implementation outcome I agree. But how did you measure acceptability in this particular study? | We agree that without formally evaluating participants’ perspectives, high acceptability cannot be directly demonstrated. In this study, we used the term to reflect the consistently high and increasing attendance from the Ministry of Health, professional associations, PORALG, R/CHMTs, and facility teams. The VMDRs fostered multi-institutional participation, expert feedback, and recommendations that showed strong commitment and willingness to collaborate across disciplines |
| 2c | How did you measure participation from leaders and experts | Attendance by representatives from professional associations, Ministry of Health, and RHMT, CHMT and facility teams was mandatory for each VMDR session, while the participation of senior leaders such as the Minister, Deputy Minister, and Permanent Secretary was occasional and not systematically tracked. Nevertheless, their presence, though infrequent, was unprecedented and signaled strong government commitment to maternal health, reinforcing accountability within the MPDSR process and motivating regional and facility teams to implement review recommendations more consistently |
| 2d | You also added fidelity and feasibility in the conclusion. These are also implementation outcomes. Kindly indicate in your methods section how all these outcomes indicated in the conclusion were measured. | Fidelity and feasibility were not the subject of this analysis. We revised the text and abstract. |
| 3 | Results: On Table 2, some of the responses are SMART eg “Commencement of weekly virtual CME sessions to improve knowledge and skills in managing common obstetric complications. A total of 42 sessions were conducted from 2022 – 2023”. However for some it is difficult to differentiate recommendations from what was actually done. | We acknowledge that what was presented was not always clear in distinguishing recommendations from actions. Our intention was to share changes and responses informed by the reviews, and as reflected in the general and specific comments in the tables, we have revised the statements to make them more specific. |
| 4 | On leadership, you indicated “The VMDRs improved leadership, participation of expert and healthcare providers and commitment to maternal health care - demonstrated by attendance of the Minister, Deputy Minister, Permanent Secretary and Chief Medical Officer, medical and nursing professional bodies, directors from MoH and PO-RALG, national, RHMTs and CHMTs, and leaders and members of professional associations. The VMDR improved the response of CHMTS and RHMTS to notification, review and implementation of action plans”. In how many of the VDMR sessions were the Minister, Deputy minister and Permanent Secretary present. Do you have a baseline on their participation in MPDSR as a comparison….in short how did you come to the conclusion that VDMR improved leadership, participation of healthcare providers. | We have revised this section to highlight the value of participation by senior leaders such as the Minister, Deputy Minister, and Permanent Secretary, who had not previously taken part in MDR sessions. Although their attendance was occasional and not systematically tracked, it was unprecedented and signaled strong government commitment to maternal health, reinforced accountability within the MPDSR process, and motivated regional and facility teams to implement review recommendations more consistently |
| 5 | The Conclusion: Does not speak to the title. The issues of acceptability as indicated earlier in 2 above | We acknowledge this observation and have revised both the conclusion and the title to better align with the study objectives, methods, and results, thereby ensuring consistency throughout the manuscript. |
| 6. | Titled changed in manuscript | Using national virtual maternal death reviews to improve the quality of care during Pregnancy, labour and birth, and Postpartum in Tanzania |

Sincerely

Dr. Ahmad M. Makuwani

Corresponding author
