# Supplementary material for: Using national virtual maternal death reviews to improve the quality of care during Pregnancy, labour and birth, and Postpartum in Tanzania
Source: PLoS One. 2026 Mar 18;21(3):e0344858. doi: 10.1371/journal.pone.0344858 (PMC12998800; doi:10.1371/journal.pone.0344858)
Supplement: Attachment — Submitted filename: Rebuttal letter .docx [file pone.0344858.s009.docx]

November 17^th^ 2025

Dear Joyce Jebet Cheptum

Academic Editor

**PLOS ONE**

Dear Editor,

On behalf of all co-authors, we thank you for the opportunity to revise our manuscript and for the constructive feedback you have provided. We carefully reviewed each comment and have revised the manuscript to address all points raised, including improvements to the introduction, methods, discussion, and reference list, as detailed in our point-by-point responses. Updated versions of the manuscript with and without tracked changes have been provided as requested. We confirm that no revisions were required to our financial disclosure statement, and therefore, it remains unchanged.

We appreciate the thoughtful guidance offered throughout this process and hope that the revised manuscript now meets the publication standards of PLOS ONE.

Point-by-point author’s response to reviewers’ comments

| **Reviewer identification** | **Comment** | **Authors’ Response** |
| --- | --- | --- |
| **Reviewer #3:** | The necessity of the study is not clearly articulated in the abstract | Thank you for the comment. We have revised the abstract to clearly explain the necessity of the study by highlighting the existing gaps in the quality of maternal death reviews in Tanzania and the need for virtual review mechanisms to strengthen review quality, accountability, and response. This provides a clearer rationale for the introduction and evaluation of VMDR. |
|  | In the methods section of the abstract, please specify the criteria for the study | Thank you for this important observation. We have revised the Methods section of the abstract to clearly specify the eligibility criteria for cases included in the analysis. The revised text now clarifies that only maternal deaths notified through the national MPDSR system and selected for VMDR, which met the WHO definition of a maternal death and had sufficient clinical documentation to determine the cause of death and contributing factors, were included. This provides greater clarity on the study population and the basis for case selection. |
|  | 3. In the last paragraph of the introduction, highlight the innovations present in your research | Thank you for the comment. We have revised the final paragraph of the Introduction to clearly articulate the innovative aspects of the VMDR model. The paragraph now explains how VMDR leverages digital platforms to enable real-time multidisciplinary review, broaden participation of national experts, strengthen accountability at regional and district levels, and link review findings to targeted mentorship and system-level actions. This revision emphasizes VMDR as an innovative advancement of the existing MPDSR process |
|  | 4. In the methods section, provide a more detailed explanation of the data collection and sampling methods | Thank you for this comment. We have revised the Methods section to clarify how cases were identified and selected, and how data were collected. The revised text now specifies that cases were drawn from the national MPDSR notification system, included only if they met the WHO definition of maternal death and had sufficient documentation, and were reviewed using the standard VMDR documentation template. We also added brief clarification on data extraction into Microsoft Excel and the deductive thematic analysis conducted. These additions provide clear and transparent description of the sampling and data collection procedure |
|  | 5.Strengthen the discussion section | Thank you for this comment. We have robustly revised the Discussion to provide clearer interpretation of the findings and to highlight how VMDR strengthens the MPDSR process by expanding specialist input, promoting shared learning and improving follow up of action plans. We also clarified the implications for clinical practice, mentorship and leadership accountability, and expanded the explanation of the proportion of anaesthesia related deaths. These revisions improve the clarity and depth of the Discussion while maintaining the original structure and key messages |
|  | 6. Many of the references are outdated; please use sources from the last five years. | Thank you for this important observation. We reviewed the entire reference list and replaced older citations where more recent evidence or updated global guidance was available, prioritising sources published between 2021 and 2025. We also retained several seminal and still-authoritative references, including ICD-MM classification, MPDSR principles, the Three Delays framework and the comfort zone theory, as these remain the primary foundations for these concepts. All updates have been reflected in both the in-text citations and the reference list |

Sincerely

Dr. Ahmad M. Makuwani

Corresponding author
