# Supplementary material for: Using national virtual maternal death reviews to improve the quality of care during Pregnancy, labour and birth, and Postpartum in Tanzania
Source: PLoS One. 2026 Mar 18;21(3):e0344858. doi: 10.1371/journal.pone.0344858 (PMC12998800; doi:10.1371/journal.pone.0344858)
Supplement: Attachment — Submitted filename: Rebuttal Jan 2026.docx [file pone.0344858.s010.docx]

5^th^ January 2026

Dear Joyce Jebet Cheptum

Academic Editor

**PLOS ONE**

Dear Editor,

On behalf of all co-authors, I would like to thank you and reviewers for a big interest shown. careful assessment of our manuscript and for the constructive feedback provided throughout the review process, including the most recent comments received on 5 January 2026. We are encouraged by the positive evaluations confirming that the manuscript is technically sound, clearly written, and supported by appropriate data and analyses. We particularly appreciate the acknowledgement that the revised manuscript has improved substantially and that all previously raised comments have been adequately addressed.

In this revision, we have carefully considered and responded to all additional comments raised, including clarifications on the national maternal mortality context, application of the three-delay model, data presentation, interpretation of regional review patterns, framing of health financing gaps, citation of comparable international experiences, and contextualisation of key findings.

Detailed point-by-point responses to each comment are provided in the accompanying response table, with a clear indication of the corresponding revisions and their locations in the manuscript. We are grateful for the opportunity to revise and strengthen the manuscript and appreciate the Editor’s and Reviewers’ guidance throughout this process.

6. **Review Comments to the Author**

| **Reviewer** | **Comment** | **Response** |
| --- | --- | --- |
| **Editor** | Dear Authors, please address the few comments related to maternal mortality in Tanzania | We have addressed this and other comments |
| **Reviewer #8:** | The author did all changes, the manuscript looks better now. Accepted for publication.All the best | Thank you very much for the positive and encouraging feedback. |
| **Reviewer #9:** | 1. What is the maternal mortality ratio for Tanzania? It did not reflect anywhere in the document. | Thank you for this important observation. Tanzania’s maternal mortality ratio is 104 maternal deaths per 100,000 live births, based on the 2022 Tanzania Demographic and Health Survey and Malaria Indicator Survey (TDHS - MIS). We acknowledge that this figure was not explicitly stated in the original version of the manuscript.  We have now revised the introduction to include the national maternal mortality ratio and appropriate citation, to better contextualize the burden of maternal mortality in Tanzania and strengthen the background of the study. |
|  | 2. Page 8. Since the three-delay model was put into consideration, how are modifiable community and family-oriented gaps that contributed to the mortality put into consideration? | Thank you for this comment. We have revised the manuscript and Figure 2 to explicitly capture client and community-related contributing factors within the application of the three-delay model. These factors accounted for 27% of the reviewed maternal deaths and include delayed care-seeking, household decision-making constraints, inadequate birth preparedness, and community - level referral challenges, as presented in the results section. |
|  | 3. Page 9. “Data not shown” How does this relate to places where the mortality occurred? What is the weighed percentage? | Thank you for pointing this. The phrase “data not shown” was included in error. All data on the place where maternal deaths occurred are fully presented in the manuscript. We have revised the text to remove this phrase and clarify the presentation accordingly |
|  | 4. Page 9 - The statement “Due to rotating schedule of region…. The rotation does not adequately explain why region with fewer death had more reviews. Is it that regions with fewer death were given more opportunity? Did the rotation favoured them more? | Thank you for this observation. We have revised the text to clarify that the higher proportion of deaths reviewed in regions with fewer maternal deaths reflects the structured rotation system, in which each region is allocated scheduled opportunities to present cases irrespective of the absolute number of deaths. As a result, regions with fewer deaths were able to review a larger proportion of their notified cases, rather than being favoured by the process. The revised text now makes this explicit to avoid misinterpretation. |
|  | 5. Page 12 – “Lack of a strong data backed investment case to advocate for financial resources in maternal healthcare “. The response documented regarding this gap seems inappropriate. Before funds can be duly allocated, there is need for adequate information or data for human resources. Allocating funds without strong data could result in inappropriate allocation, | Thank you for this important clarification. We agree that allocation of financial resources should be guided by robust data and not precede it. We have revised the text to clarify that VMDR findings strengthened the data-backed investment case for maternal health by generating actionable evidence on gaps in human resources, competencies, and system readiness, which then informed prioritisation and allocation of resources. The revised text now emphasises evidence-informed decision-making rather than allocation in the absence of data. |
|  | 6. Page 16.. “Similar benefits have been observed in other virtual and digital review initiatives”. Can the countries be cited and referenced? | Thank you for this comment. We have revised the manuscript to explicitly cite and reference the countries where similar virtual and digital review initiatives have been implemented. The revised text now specifies countries involved in international virtual confidential reviews and contextualises telehealth - supported maternal health initiatives in low-resource settings, with appropriate references added in the discussion section. |
|  | 7. Page 17 “ Most maternal deaths reviewed through VMDR occurred in health facilities and were linked to preventable or modifiable factors, including gaps in clinical knowledge, delayed recognition of complications, inconsistent adherence to standards of care, and inadequate supervision”. Can the context be included? For example, “From review, most maternal death…………”. It will help establish the statement as a context for comparison with Tanzania. | Thank you for this helpful suggestion. We have revised the sentence to explicitly situate the statement within the VMDR review context in Tanzania, clarifying that the observation is based on findings from the reviewed cases. The revised wording now begins with “From the VMDR review process in Tanzania…”, thereby establishing a clear contextual basis for comparison with findings from other settings. |

Sincerely

Ahmad M. Makuwani

**Corresponding author**
